# Label-Free Phenotypic Profiling Identified D-Luciferin as a GPR35 Agonist

**DOI:** 10.1371/journal.pone.0034934

**Published:** 2012-04-12

**Authors:** Haibei Hu, Huayun Deng, Ye Fang

**Affiliations:** Biochemical Technologies, Science and Technology Division, Corning Inc., Corning, New York, United States of America; German Cancer Research Center, Germany

## Abstract

Fluorescent and luminescent probes are essential to both *in vitro* molecular assays and *in vivo* imaging techniques, and have been extensively used to measure biological function. However, little is known about the biological activity, thus potential interferences with the assay results, of these probe molecules. Here we show that D-luciferin, one of the most widely used bioluminescence substrates, is a partial agonist for G protein-coupled receptor-35 (GPR35). Label-free phenotypic profiling using dynamic mass redistribution (DMR) assays showed that D-luciferin led to a DMR signal in native HT-29 cells, whose characteristics are similar to those induced by known GPR35 agonists including zaprinast and pamoic acid. DMR assays further showed that D-luciferin is a partial agonist competitive to several known GPR35 agonists and antagonists. D-luciferin was found to cause the phosphorylation of ERK that was suppressed by known GPR35 antagonists, and also result in β-arrestin translocation signal but with low efficacy. These results not only suggest that D-luciferin is a partial agonist of GPR35, but also will evoke careful interpretation of biological data obtained using molecular and *in vivo* imaging assays when these probe molecules are used.

## Introduction

With the advances in detection technology have expanded the applications of fluorescent and luminescent probe molecules for measuring a myriad of biological functions [1]. However, these probe molecules could introduce assay artifacts and alter performance or functions of proteins and cells. Further, dominated in the target-centric basic research and drug discovery are molecular assays that measure a specific signaling pathway and/or molecule to infer the functional consequences of drugs and molecules [2], [3]. However, these molecular assays often use artificial systems and fluorescent and luminescent molecules, and are limited to a predetermined mechanism of action (MoA) by measuring a single signaling molecular species one at a time [4], [5]. Label-free cellular assays including dynamic mass redistribution (DMR) assays enabled by optical biosensors have emerging an attractive alternative to delineate receptor biology and drug pharmacology at the whole level [6]–[10]. Label-free assays are non-invasive with high sensitivity, so it is possible to investigate the vectorial behaviors of receptors and molecules including fluorescent and luminescent molecules in native cells. Further, label-free assays offer an integrated functional cellular response, so it is possible to cover a diverse range of pathways downstream a receptor [11], and to detect ligands of diverse MoAs for a target receptor [12].

**Figure 1:**
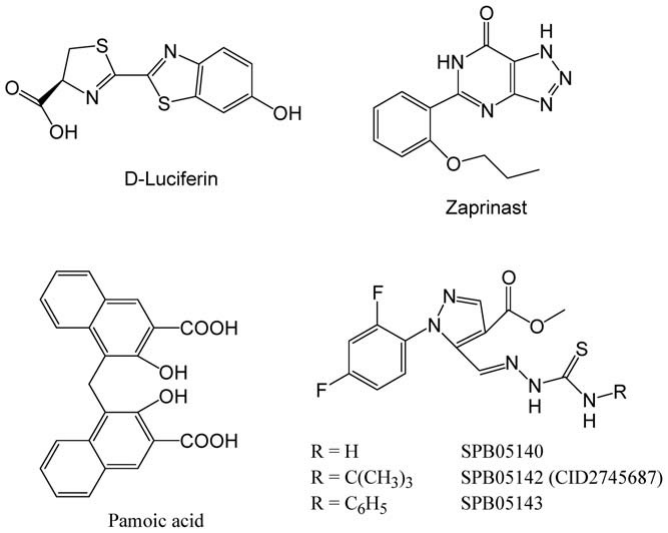
Chemical structures of GPR35 ligands. The ligands studied are D-luciferin, zaprinast, pamoic acid, SPB05140, SPB05142 and SPB05143.

Firefly luciferin or D-luciferin ((S)-2-(6′-hydroxy-2′-benzothiazolyl)thiazoline-4-carboxylic acid) (**Fig. 1**) belongs to a class of light-emitting molecules utilized by a luciferase or photoprotein in the cells of various bioluminescent organisms [13]. D-luciferin is the natural substrate of luciferase responsible for the characteristic yellow light emission from fireflies. D-luciferase catalyzes a bioluminescence reaction that uses luciferin, Mg-ATP and molecular oxygen to produce an electronically excited oxyluciferin species, emitting light with a broad emission spectrum and a peak at ≈560 nm yellow-green light [14]. Given the high quantum yield of the luciferin-luciferase reaction [15] and the change in bioluminescence color caused by subtle structural differences in luciferase [16], D-luciferin has been widely used as a substrate to monitor luciferase activity for *in vitro* assays and an optical imaging agent for *in vivo* imaging [17]. Thus, we were interested in examining the pharmacological activity of D-luciferin. Here we applied DMR assays to characterize the activity of D-luciferin in native human colon adenocarcinoma grade II cell line HT-29, and found that D-luciferin is a partial agonist of G protein-coupled receptor (GPCR)-35 (GPR35), a poorly characterized orphan GPCR.

## Results

We used four different types of assays to ascertain the agonist activity of D-luciferin at GPR35. First, multiple DMR assays were employed to characterize the pharmacology of D-luciferin in HT-29 cells, a native cell line endogenously expressing GPR35 [18]. DMR agonist assay was first used to detect the DMR signal induced by D-luciferin. DMR antagonist assay was then used to determine the specificity of the D-luciferin-induced DMR to the activation of endogenous GPR35 using SPB05142, a known GPR35 antagonist with moderate potency [19], [20]. DMR desensitization assay was then used to confirm the desensitization of GPR35 to the repeated stimulation with known GPR35 agonists after D-luciferin treatment. DMR co-stimulation assay was finally used to determine the competitive agonism of D-luciferin with pamoic acid, a known GPR35 agonist [20]. Second, D-luciferin-induced internalization of endogenous GPR35 in HT-29 cells was examined. Third, D-luciferin-induced ERK phosphorylation in the absence and presence of GPR35 antagonists was examined in HT-29 cells. Fourth, Tango β-arrestin translocation assay was finally employed to examine the agonism of D-luciferin to cause β-arrestin translocation in an engineered cell line.

### D-luciferin triggered a robust DMR signal in HT-29

**Figure 2:**
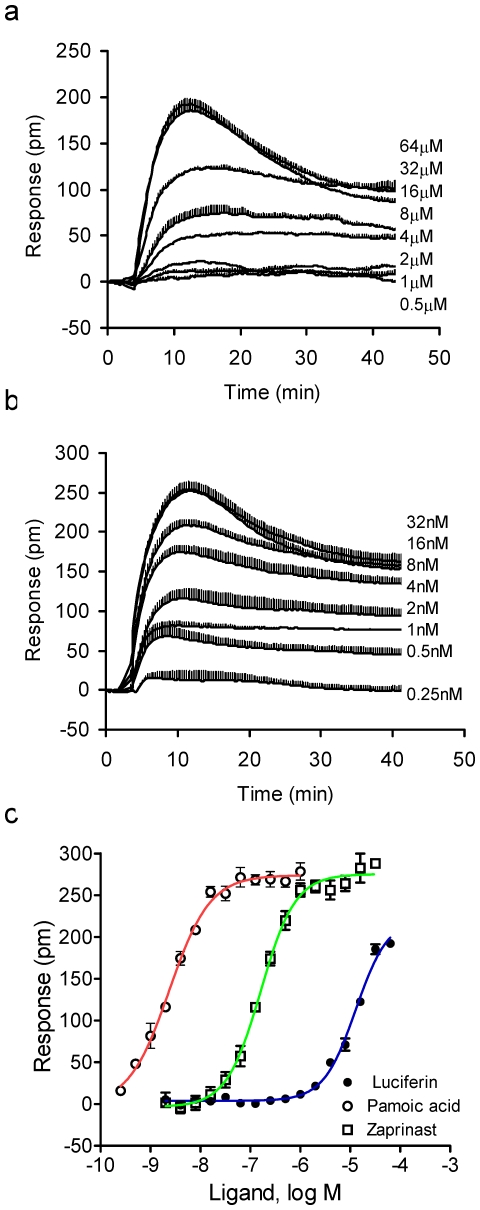
DMR characteristics of ligands in HT-29 cells. (a) The dose-dependent kinetic DMR signals induced by D-luciferin; (b) the dose-dependent kinetic DMR signals induced by pamoic acid; and (c) the P-DMR amplitudes of three ligands as a function of ligand dose. All data represent mean ± s.d. from 2 independent measurements, each in duplicate (n = 4), except for zaprinast (n = 8).

We first characterized the activity of D-luciferin in HT-29 using DMR agonism assay, which directly measure its agonist activity. Results showed that D-luciferin led to a dose-dependent and saturable DMR signal (**Fig. 2a**). Its DMR is biphasic, consisting of an early positive DMR (P-DMR) event and a late negative-DMR (N-DMR) event. The N-DMR event eventually decays back to a steady elevated level, compared to the baseline. Based on its P-DMR amplitude, the D-luciferin DMR dose-response fitted well with a monophasic sigmoidal curve, leading to an EC_50_ of 12.9±1.5 µM (2 independent measurements, each in duplicate, n = 4). Furthermore, titration of D-luciferin up to 128 µM in the absence of cells showed that it led to a negligible DMR signal, suggesting that the D-luciferin-induced DMR in HT-29 cells is specific to the cellular response.

Our recent studies using DMR phenotypic profiling have led to discovery of novel GPR35 agonists including tyrphostin analogs [18], 2-(4-methylfuran-2(5H)-ylidene)-malononitrile and thieno[3,2-b]thiophene-2-carboxylic acid derivatives including YE210 [19]. We also found that the negative charge groups such as carboxylic acid and malononitriles appear to be essential to activate endogenous GPR35 in HT-29 cells [19]. Since D-luciferin also consists of a thiazoline-4-carboxylic acid moiety and its DMR characteristics are similar to those induced by the known GPR35 agonists including zaprinast [18] and pamoic acid (**Fig. 2b**), we speculated that D-luciferin is a novel agonist for GPR35. Comparing to the DMR of D-luciferin, both zaprinast and pamoic acid gave rise to higher potency with an EC_50_ rank order of pamoic acid (2.4±0.4 nM, n = 4)<zaprinast (162.1±9.7 nM, n = 4)<D-luciferin, as well as higher efficacy based on their maximal P-DMR amplitude with an rank order of pamoic acid (274±11 pm, picometer in shift of the resonant wavelength)∼zaprinast (276±17 pm)>D-luciferin (198±12 pm) (n = 36) (**Fig. 2c**). These results suggest that D-luciferin is a partial agonist with a relatively low potency for GPR35.

### D-Luciferin is competitive to known GPR35 ligands to activate GPR35

**Figure 3:**
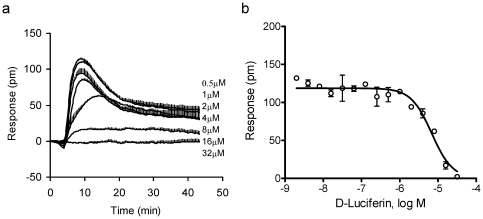
The desensitization of HT-29 to 250 nM zaprinast after 1 hr pretreatment with D-luciferin at different doses. Only the zaprinast-induced DMR signals were shown: (a) the real time kinetic response of zaprinast; and (b) the zaprinast P-DMR amplitudes as a function of D-luciferin concentration. All data represent mean ± s.d. from 2 independent measurements, each in duplicate (n = 4).

Next, we examined the specificity of D-luciferin to GPR35. First, we performed DMR desensitization assay, where the cells were pre-stimulated with D-luciferin at different doses for 1 hr, followed by repeated stimulation with zaprinast at a fixed dose. Results showed that D-luciferin dose-dependently caused HT-29 cells desensitized to the repeated stimulation with 250 nM zaprinast, leading to an apparent IC_50_ of 6.8±0.7 µM (2 independent measurements, each in duplicate, n = 4) (**Fig. 3**), which was close to the EC_50_ of D-luciferin to trigger its DMR signal. This result suggests that both D-luciferin and zaprinast activate the same receptor, GPR35.

**Figure 4:**
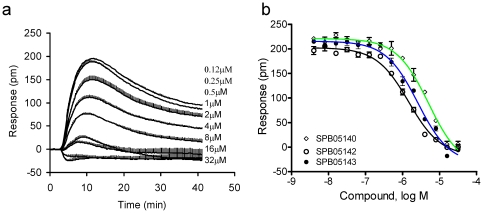
The blockage of the D-luciferin-induced DMR by three GPR35 antagonists. Only the D-luciferin DMR signals were shown: (a) the real time kinetic response of D-luciferin; and (b) the D-luciferin P-DMR amplitudes as a function of GPR35 antagonist concentration. The three GPR35 antagonists were SPB05140, SPB05142 and SPB05143. All data represent mean ± s.d. from 2 independent measurements, each in duplicate (n = 4).

Second, we performed DMR antagonism assay, where the cells were pre-stimulated with a known GPR35 antagonist at different doses for 5 min, followed by the stimulation with D-luciferin at a fixed dose. Previously we had showed that the known GPR35 antagonist CID2745687 (SPB05142; methyl-5-[(tert-butylcarbamothioylhydrazinylidene)-methyl]-1-(2,4-difluorophenyl)-pyrazole-4-carboxylate) (**Fig. 1**) up to 64 µM did not lead to any detectable DMR in HT-29 cells, but almost completely blocked the DMR induced by zaprinast [19]. Its two analogs, SPB05140 and SPB05143, up to 64 µM also triggered little response in HT-29. However, all three compounds dose-dependently blocked the DMR induced by D-luciferin at 32 µM (**Fig. 4a**); and their apparent IC_50_ values were 5.0±0.9 µM, 1.5±0.2 µM, and 2.6±0.3 µM for SPB05140, SPB05142 and SPB05143, respectively (2 independent measurements, each in duplicate, n = 4) (**Fig. 4b**). It is worthy noting that the presence of SPB05142 at the two highest doses examined (16 and 32 µM) converted the D-luciferin DMR to a small but detectable negative DMR signal, suggesting that D-luciferin may also activate another cellular target beside GPR35. Nonetheless, these results suggest that all three compounds are antagonists for GPR35, and D-luciferin is a GPR35 agonist.

**Figure 5:**
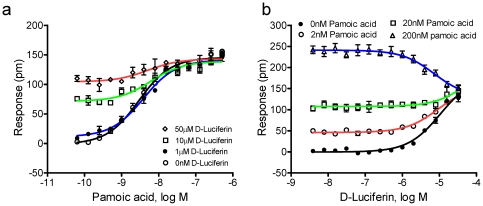
The DMR arising from co-stimulation with two agonists. (a) The dose-dependent responses of pamoic acid in the absence and presence of D-luciferin at three different fixed doses; the cell seeding density was 25000 cells per well; and (b) the dose-dependent response of D-luciferin in the absence and presence of pamoic acid at three fixed doses, the cell seeding density was 32000 cells per well. The ligand P-DMR amplitudes were used to calculate all dose responses. All data represent mean ± s.d. from 2 independent measurements, each in duplicate (n = 4).

Third, we performed DMR co-stimulation assays, where concentration-response curves to pamoic acid were performed in the presence of different fixed doses of D-luciferin; *vice versa*, the dose response curves to luciferin were done in the presence of different fixed doses of pamoic acid. The presence of D-luciferin at different fixed doses did not alter the potency of pamoic acid, but increased the starting signal in a dose-dependent manner. Furthermore, pamoic acid still resulted in an increased signal in a dose-dependent fashion when D-luciferin up to its saturating dose (50 µM) was presented, and its maximal responses remained the same level as the highest dose of pamoic acid in the absence of D-luciferin (**Fig. 5a**). Conversely, for low doses of pamoic acid (2 and 20 nM), D-luciferin dose-dependently led to an increased DMR signal; however, for pamoic acid at 200 nM (a saturating concentration), high doses of D-luciferin reduced the DMR in a dose-dependent manner (**Fig. 5b**). Furthermore, the maximal responses of pamoic acid in the presence of D-luciferin at all three doses reached the same level as the highest concentrations of D-luciferin in the absence of pamoic acid. These results are best explained by different efficacy of the two compounds interacting at an overlapping binding site, and D-luciferin is less efficacious agonist of GPR35 than pamoic acid. Taken together, these DMR assays suggest that D-luciferin is a partial agonist for GPR35.

### D-Luciferin is a partial agonist of GPR35

**Figure 6:**
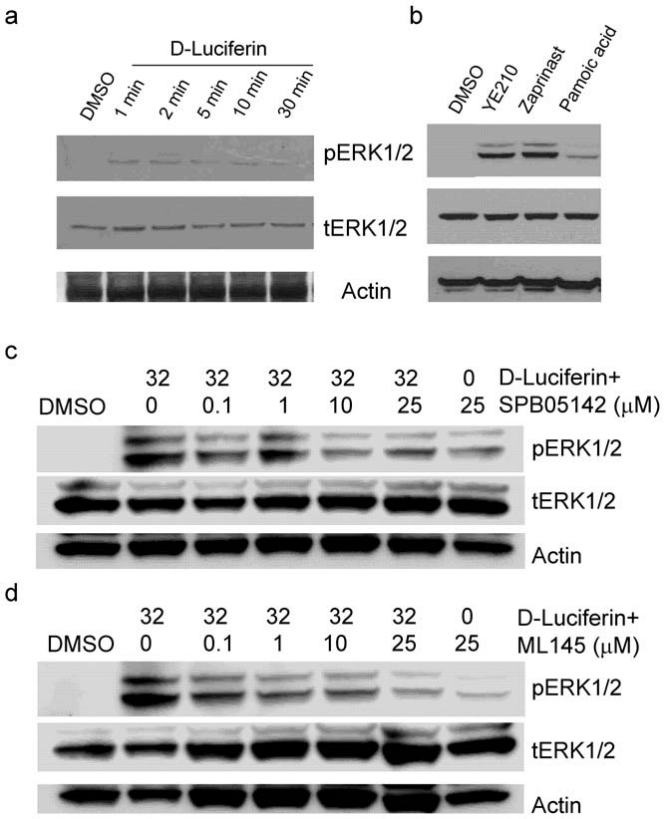
D-Luciferin triggered ERK phosphorylation via GPR35. (a) Western blot of phosphorylated ERK1/2 (pERK1/2) after treated with 32 µM D-luciferin for different times; (b) Western blot of phosphorylated ERK 30 min after treated with three different known GPR35 agonists, YE210 (1 µM), zaprinast (1 µM) and pamoic acid (1 µM); (c) Western blot of phosphorylated ERK 5 min after treated with 32 µM in the presence of SPB05142 at different doses; (d) Western blot of phosphorylated ERK 5 min after treated with 32 µM in the presence of ML145 at different doses. The phosphorylated ERK and total ERK were blotted, and actin was used as control. Representative images obtained from 2 independent measurements were used.

Since ligand-directed functional selectivity or biased agonism is quite common to many, if not all, GPCRs [21], we next examined the activity of D-luciferin using ERK phosphorylation, receptor internalization and Tango β-arrestin translocation assays. ERK phosphorylation is a hallmark of the activation and signaling of many GPCRs including GPR35 [20]. Results showed that stimulation of HT-29 cells with D-luciferin led to phosphorylation of ERK in a time-dependent manner, and the maximum ERK phosphorylation was observed 2 min after stimulation (**Fig. 6a**). As controls, the two known GPR35 agonists YE210 and zaprinast also led to ERK phosphorylation, but pamoic acid led to much lower ERK phosphorylation (**Fig. 6b**). The two GPR35 antagonists, SPB05142 and ML145 [22], dose-dependently attenuated the D-luciferin-induced ERK phosphorylation (**Fig. 6c and d**). At the high dose (25 µM) examined, SPB05142 alone led to a weak but detectable ERK phosphorylation via an unknown mechanism. These results suggest that D-luciferin resulted in ERK phosphorylation via the activation of GPR35.

**Figure 7:**
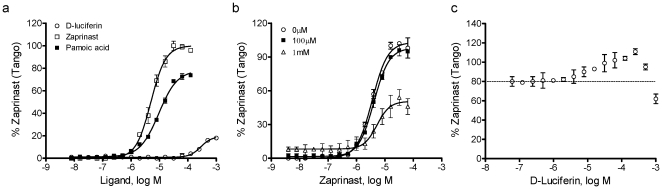
D-Luciferin caused β-arrestin translocation via GPR35. (a) The dose-dependent responses of three ligands as measured using Tango™ β-arrestin translocation gene reporter assays. (b) The dose-dependent responses of zaprinast in the presence of D-luciferin at different fixed doses. (c) The dose-dependent responses of D-luciferin in the presence of 10 µM zaprinast. All data were normalized to the zaprinast maximal response. The data represents mean ± s.d. from two independent measurements, each in duplicate (n = 4).

Tango™ GPR35 β-arrestin assay showed that zaprinast led to a dose dependent response in Tango™ GPR35-*bla* U2OS cells with an EC_50_ of 5.0±0.4 µM (2 independent measurement, each in duplicate, n = 4) (**Fig. 7a**). Similarly, pamoic acid was also active in this assay with an EC_50_ of 9.4±0.7 µM but a lower efficacy (2 independent measurement, each in duplicate, n = 4). Compared to DMR assays, Tango β-arrestin translocation assay resulted in an obvious right-shift in potency, and such a shift was more significant for pamoic acid (∼3800 fold) than zaprinast (∼37 fold). Similarly, D-luciferin also triggered clear β-arrestin translocation signal with a relatively low potency (EC_50_, 277±25 µM; 2 independent measurements, n = 4), and low efficacy, as evidenced by that its maximal response was only 20% of the zaprinast maximal response (**Fig. 7a**). In a Tango co-stimulation assay the presence of 100 µM D-luciferin only slightly suppressed the maximal response of zaprinast; however, the presence of 1 mM D-luciferin not only increased the starting signal but also suppressed the maximal response of zaprinast (**Fig. 7b**). Further, the presence of D-luciferin at the two different doses had little impact on the potency of zaprinast. Furthermore, in an alternative Tango co-stimulation assay wherein zaprinast was fixed at 10 µM, a dose close to its EC_80_, D-luciferin triggered a bi-phasic dose response – it initially increased the Tango response, but at high doses it started to suppress the zaprinast responses (**Fig. 7c**). These results were best explained by that zaprinast is a full agonist of GPR35, D-luciferin is a partial agonist competitive to zaprinast but with much lower potency.

**Figure 8:**
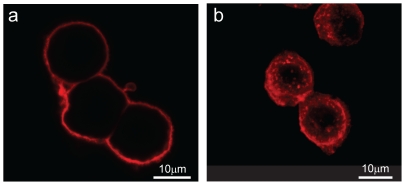
D-Luciferin triggered internalization of endogenous GPR35 in HT-29 cells. Representative confocal fluorescence images of HT-29 under different conditions: (**a**) treated with the assay vehicle containing 0.1% DMSO; (**b**) treated with 32 µM D-luciferin. The images were obtained after compound treatment for 1 hr, permeabilized, stained with anti-GPR35, followed by fluorescent secondary antibody. Red: GPR35 stains. Representative images obtained from 2 independent measurements were used.

Finally, receptor internalization assays showed that similar to zaprinast and tyrphostin-51 [18], D-luciferin resulted in remarked internalization of endogenous GPR35 in HT-29 cells (**Fig. 8**). Together, these results further strengthen our conclusions; that is, D-luciferin is a partial agonist for GPR35.

## Discussion

Fluorescent and luminescent probe molecules have been widely used as a substrate or a label for both *in vitro* molecular assays and *in vivo* biomedical imaging. However, little is known for their activity in cells and tissues. This is partly due to the relatively low sensitivity and single endpoint measurements of conventional assays, and partly due to the interference of these labels with the measurements themselves. Together with their insensitivity to colors of ligands, the wide pathway coverage makes label-free cellular assays an attractive means to detect many different classes of ligands, each having their own biased agonism, for a given target. D-luciferin is one of the most widely used bioluminescent substrates in measuring a myriad of biological functions. Here we employed label-free cellular assays to characterize the activity of D-luciferin, and discovered a previously unknown activity of D-luciferin; that is, D-luciferin is a partial agonist of GPR35.

GPR35 is an orphan GPCR, and has been implicated in hypertension [23], coronary artery disease [24], asthma [25], pain [26], inflammation [27], and cancers [28]. Several different classes of molecules have been discovered to modulate GPR35 activity [18], [19], [21], [29]–[33]. These GPR35 ligands exhibit quite diversity in chemical scaffold, making one speculate the mostly unknown biology and pathophysiology of GPR35 [34]. Nonetheless, the discovery of D-luciferin to be a partial agonist of GPR35 has added a novel chemical class to the increasing list of pharmacological tools available for probing the biology and pathophysiology of GPR35. Furthermore, we found that the potency of D-luciferin to activate GPR35 in native HT-29 is comparable to it K_m_ value (16 µM) binding to purified luciferase enzyme [13]. Considering that the doses used for *in vitro* assays and *in vivo* imaging are often much higher that its K_m_ [13], the GPR35 agonist activity of D-luciferin observed here will call close examination of the pharmacological activity of fluorescent and luminescent probe molecules widely used in conventional assays, and also evoke careful interpretation of biological data when these molecules are used.

## Methods

### Materials and cells

Pamoic acid and D-luciferin were obtained from Sigma Chemical Co. (St. Louis, MO, USA). ML145 was obtained from Tocris Chemical Co. (St. Louis, MO, USA). Zaprinast was purchased from Enzo Life Sciences (Plymouth Meeting, PA). SP05140, SPB05142 and SPB05143 were obtained from Ryan Scientific, Inc. (Mt. Pleasant, SC). YE210 [19] and Epic® 384-well biosensor microplates were obtained from Corning Inc. (Corning, NY). Mouse monoclonal anti-phosphorylated extracellular-signal-regulated kinase 1/2 (anti-pERK1/2) (Cat#M9682) and mouse monoclonal anti-ERK1/2 (#M7431) were obtained from Sigma.

HT-29 was obtained from American Type Cell Culture (Manassas, VA). The cells were cultured in McCoy's 5a Medium Modified supplemented with 10% fetal bovine serum, 4.5 g/liter glucose, 2 mM glutamine, and antibiotics at 37°C under air/5% CO_2_. Tango™ GPR35-bla U2OS cells were purchased from Invitrogen (Grand Island, NY). Tango™ GPR35-*bla* U2OS cells stably expresses human GPR35 linked to a TEV protease site and a Gal4-VP16 transcription factor, as well as a β-arrestin/TEV protease fusion protein and the β-lactamase reporter gene under the control of a UAS response element. The cells were cultured according to the protocols recommended by the supplier. Briefly, the cells were passed using McCoy's 5A medium (Invitrogen, Cat#16600-082) supplemented with 10% dialyzed fetal bovine serum, 0.1 µM NEAA, 25 µM Hepes (pH 7.3), 1 mM sodium pyruvate, 100 U/ml penicillin, 100 µg/ml streptomycin, 200 µg/ml zeocin, 50 µg/ml hygromycin, and 100 µg/ml geneticin in a humidified 37°C/5% CO_2_ incubator.

### Dynamic mass redistribution assays

All DMR assays [35] were performed using Epic® system (Corning Inc.). Epic® is a wavelength interrogation reader system tailored for resonant waveguide grating biosensors in microtiter plates. This system consists of a temperature-control unit (26°C), an optical detection unit, and an on-board liquid handling unit with robotics. The detection unit is centered on integrated fiber optics, and enables kinetic measures of cellular responses with a time interval of ∼15 sec. Cells were directly seeded in Epic® plates and cultured overnight to form confluent monolayer in the cell culture medium. The cell seeding density was 32, 000 cells per well, unless specifically mentioned. After being washed twice, the cells were maintained with HBSS (1× Hanks balanced salte buffer, 10 mM Hepes-KOH, pH 7.1) and further incubated inside the system for 1 hr. After a 2-min baseline, the agonism profiles of ligands in the absence and presence of another compound at a fixed dose were recorded immediately after the ligand additions, while the antagonism or desensitization profiles were indicated by the DMR of a ligand at its EC_50_ or EC_100_ after the cells were pretreated with a compound or an agonist, respectively, for 1 hr. The assay vehicle containing equal amount of DMSO (dimethyl sulfoxide) was used as a control. All ligand-induced DMR were background corrected. All EC_50_ or IC_50_ reported were calculated based on the amplitudes of DMR signals at 8 min post agonist stimulation, unless specifically mentioned.

### ERK MAPK assays

The p44/42 MAP kinases were examined using Western blotting. Whole cell lysates were collected after the cells were treated with a compound or DMSO for 1 hr. Equivalent gel loading was confirmed by probing with anti-actin body. The total ERK1/2 and phosphorylated ERK1/2 were blotted using respective antibodies.

### Tango™ β-arrestin assays

Tango™ GPR35 β-arrestin assay utilizes GPR35 agonist-induced recruitment of protease tagged β-arrestin to GPR35 that has been modified at the C-terminus to include a transcription factor linked by a protease cleavage site. As a result of arrestin recruitment, the protease cleaves the transcription factor from the receptor, which then translocates to the nucleus and activates the expression of β-lactamase. The assay protocol recommended by the supplier was used. Briefly, 10000 cells per well were seeded in 384-well, black-wall, clear bottom assay plates with low fluorescence background (Corning), and cultured in DMEM (Invitrogen, 10569-010) supplemented with 10% dialyzed fetal bovine serum, 0.1 µM NEAA, 25 µM Hepes (pH 7.3), 100 U/ml Penicillin, and 100 µg/ml Streptomycin. After overnight culture, the cells were stimulated with ligands for 5 hrs in a humidified 37°C/5% CO_2_, and then loaded with the cell permeable LiveBLAzer™ FRET B/G substrate. After the two hour incubation the coumarin∶fluorescein ratio in the presence of a ligand was measured using Tecan Safire II microplate reader (Männedorf, Switzerland). All results obtained were normalized to the zaprinast maximal responses using an intra-plate referencing protocol; that is, a dose response of zaprinast was obtained within the same plate, and a compound response was then normalized to the maximal response of zaprinast which was set to be 100%.

### Receptor internalization

HT-29 cells were plated on an 8-well chamber slide (Nalge Nunc International, Rochester, NY, USA) with a seeding density of 10,000 cells per well and incubated at 37°C for 24 hrs. Next day, cells were stimulated with a compound or equal amount of DMSO at 37°C for 1 hr. Afterwards, cells were fixed with 4% formaldehyde in 1×PBS for 15 min, followed by blocking and permeabilization in a buffer containing 4% goat serum, 0.1% bovine serum albumin (BSA), 0.1% Triton ×100 in 1×PBS for 2 hrs. After 5 min wash with PBS, fixed cells were incubated with the anti-GPR35 (1∶500) (anti-GPR35, T-14, intracellular domain) (Santa Cruz biotechnology, Santa Cruz, CA, USA) in 3% BSA/PBS buffer for 24 hrs, followed by incubation with secondary antibody Alexa Fluor® 594 donkey anti-goat IgG (H+L) (1∶500) (Invitrogen) in 3% BSA/PBS for 1 hr at room temperature. Cells were finally washed once with PBS and sealed with 1.5 mm thick glass cover-slip (Corning, NY). Dried slides were stored at 4°C until imaging. Confocal imaging was performed with Zeiss confocal microscope Axiovert 40. The specificity of anti-GPR35 was confirmed by the control peptide from the supplier. Staining showed that the control peptide completely blocked the staining of HT-29 cells with the anti-GPR35 antibody. The collected images were analyzed using MacBiophotonics Image J software (http://www.macbiophotonics.ca/downloads.htm).

### Statistical analysis

For dose responses, at least two independent measurements, each done at least in duplicate, were performed to calculate the mean responses and the standard deviations (s.d.).
